# Cells Derived from Human Long Bone Appear More Differentiated and More Actively Stimulate Osteoclastogenesis Compared to Alveolar Bone-Derived Cells

**DOI:** 10.3390/ijms21145072

**Published:** 2020-07-17

**Authors:** Cindy Kelder, Cornelis J. Kleverlaan, Marjolijn Gilijamse, Astrid D. Bakker, Teun J. de Vries

**Affiliations:** 1 Department of Oral Implantology and Prosthodontics, Academic Centre For Dentistry Amsterdam (ACTA), University of Amsterdam and Vrije Universiteit Amsterdam, Gustav Mahlerlaan 3004, 1081 LA Amsterdam, The Netherlands; 2 Department of Oral Cell Biology, Academic Centre For Dentistry Amsterdam (ACTA), University of Amsterdam and Vrije Universiteit Amsterdam, Amsterdam Movement Sciences, Gustav Mahlerlaan 3004, 1081 LA Amsterdam, The Netherlands; a.bakker@acta.nl; 3 Department of Dental Material Sciences, Academic Centre For Dentistry Amsterdam (ACTA), University of Amsterdam and Vrije Universiteit Amsterdam, Gustav Mahlerlaan 3004, 1081 LA Amsterdam, The Netherlands; c.kleverlaan@acta.nl; 4 Department of Oral and Maxillofacial Surgery and Oral Pathology, Amsterdam UMC, Location VUmc, Vrije Universiteit, and ACTA, University of Amsterdam and Vrije Universiteit, Gustav Mahlerlaan 3004, 1081 LA Amsterdam, The Netherlands; m.gilijamse@amsterdamumc.nl or; 5 Department of Oral and Maxillofacial Surgery, OLVG, 1081 LA Amsterdam, The Netherlands; 6 Department of Periodontology, Academic Centre For Dentistry Amsterdam (ACTA), University of Amsterdam and Vrije Universiteit Amsterdam, Amsterdam Movement Sciences, Gustav Mahlerlaan 3004, 1081 LA Amsterdam, The Netherlands

**Keywords:** jaw bone, vitamin D_3_, calcitriol, cell differentiation, osteoblasts, osteoclasts

## Abstract

Osteoblasts derived from mouse skulls have increased osteoclastogenic potential compared to long bone osteoblasts when stimulated with 1,25(OH)_2_ vitamin D_3_ (vitD_3_). This indicates that bone cells from specific sites can react differently to biochemical signals, e.g., during inflammation or as emitted by bioactive bone tissue-engineering constructs. Given the high turn-over of alveolar bone, we hypothesized that *human* alveolar bone-derived osteoblasts have an increased osteogenic and osteoclastogenic potential compared to the osteoblasts derived from long bone. The osteogenic and osteoclastogenic capacity of alveolar bone cells and long bone cells were assessed in the presence and absence of osteotropic agent vitD_3_. Both cell types were studied in osteogenesis experiments, using an osteogenic medium, and in osteoclastogenesis experiments by co-culturing osteoblasts with peripheral blood mononuclear cells (PBMCs). Both osteogenic and osteoclastic markers were measured. At day 0, long bones seem to have a more late-osteoblastic/preosteocyte-like phenotype compared to the alveolar bone cells as shown by slower proliferation, the higher expression of the matrix molecule *Osteopontin* (*OPN)* and the osteocyte-enriched cytoskeletal component *Actin alpha 1* (*ACTA1)*. This phenotype was maintained during the osteogenesis assays, where long bone-derived cells still expressed more *OPN* and *ACTA1*. Under co-culture conditions with PBMCs, long bone cells also had a higher *Tumor necrose factor-alfa* (*TNF-α*) expression and induced the formation of osteoclasts more than alveolar bone cells. Correspondingly, the expression of osteoclast genes *dendritic cell specific transmembrane protein* (*DC-STAMP*) and *Receptor activator of nuclear factor kappa-Β ligand (RankL)* was higher in long bone co-cultures. Together, our results indicate that long bone-derived osteoblasts are more active in bone-remodeling processes, especially in osteoclastogenesis, than alveolar bone-derived cells. This indicates that tissue-engineering solutions need to be specifically designed for the site of application, such as defects in long bones vs. the regeneration of alveolar bone after severe periodontitis.

## 1. Introduction

One of the great challenges of periodontal treatment is the regeneration of the tooth-anchoring degraded alveolar bone. Surprisingly, little is known about both the osteogenic and osteoclastogenic capacity of the cells retrieved from this skeletal site. Bone tissue-engineering approaches using scaffolds are mostly based on research on long bones, whereas the restoration of alveolar bone between teeth after periodontal treatment is promoted by the cells derived from these unique alveolar bone structures. Ideally, the cells in the bone surrounding a tissue engineering construct are instructed by the signals emitted by the constructs to form bone, and also send cues to recruit stem cells, which can differentiate into cells that form bone. Cells from different skeletal sites may react differently to biological signals, e.g., as released by tissue-engineering constructs, such as vitD_3_ [1]. Furthermore, communication with other cell types might also be different. The reason why these cells may behave differently might lie in their origin, as alveolar bone originates from neural crest cells while long bone originates from the mesoderm [2].

An indication that cells from different skeletal sites may be different is shown by the in vitro comparison of orofacial and iliac crest *human* bone marrow stromal cells (hMSCs), where orofacial stromal cells were shown to have a higher proliferation and express higher levels of alkaline phosphatase (ALP), while the cells from the iliac crest responded more to osteogenic and adipogenic cues [3]. Another indication that there are differences between the cells from different skeletal sites is the cells’ responsiveness to biological components and drugs. In response to bone morphogenetic protein 2 (BMP-2), orofacial hMSCs have a higher expression of osteogenic markers such as ALP and *OPN* than the cells from adult iliac crest [4]. Lastly, bisphosphonates are used to prevent the loss of bone density by reducing osteoclastic bone resorption and are therefore prescribed for patients with osteoporosis [5]. The fact that longer and higher treatment with bisphosphonates can lead to the necrosis of the jaw [6,7] and atypical femoral fractures [8,9] indicate that their effect on long bone differs from that on alveolar bone. These different responses indicate that for the most optimal effect, bone tissue-engineering constructs need to be developed site specifically.

For the remodeling of bone, both osteoblast and osteoclast are needed, where the osteoblasts form bone and osteoclasts resorb bone [10]. Several studies also show that osteoclasts differ between skeletal sites. Mouse osteoblasts from calvaria lead to a higher number of osteoclasts compared to osteoblast derived from long bones [1], and De Souza Faloni et al. show that marrows from mice, derived from the jaw and long bone, have different osteoclastogenic potential [11]. This indicates that for optimal bone remodeling, the different effects of constructs on oseoclastogenesis also need to be considered and may also differ per skeletal site. Knowledge of local cells, such as the stem cells of the oral cavity [12], will ultimately lead to the better healing and osseointegration of implants [13].

Biological components can be used to enhance bone tissue-engineering constructs. Moreover, vitD_3_ is a such biological component often used in bone tissue engineering as it can promote the osteogenic differentiation of hMSCs [14]. Moreover, vitD_3_ also affects the osteoclastogenic differentiation of osteoblasts in vitro by inducing the *RankL* expression [15]. We recently demonstrated that the way vitD_3_ is administered, mimicking release from tissue-engineering constructs, and affecting the osteogenic capacity of adipose tissue-derived mesenchymal stem cells [16].

In the present study, we compared the degree of differentiation of bone cells derived from *human* alveolar and long bones, their production of signaling molecules, and their ability to stimulate osteoclast formation in the presence or absence of the biological component vitD_3_. We hypothesize that alveolar bone cells have an increased osteogenic and osteoclastogenic potential compared to long bone cells, as alveolar bone cells seem to have a higher turnover, and therefore, we expect less differentiated cells.

## 2. Results

### 2.1. Alveolar Bone Cells and Long Bone Cells Differ in Basic Appearance, Proliferation, and Expression of Mature Bone Cell Markers

**Figure 1:**
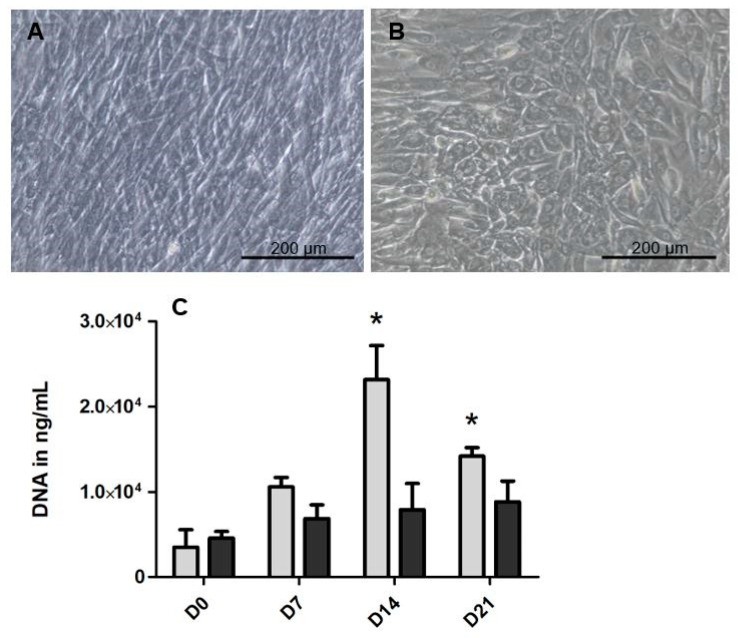

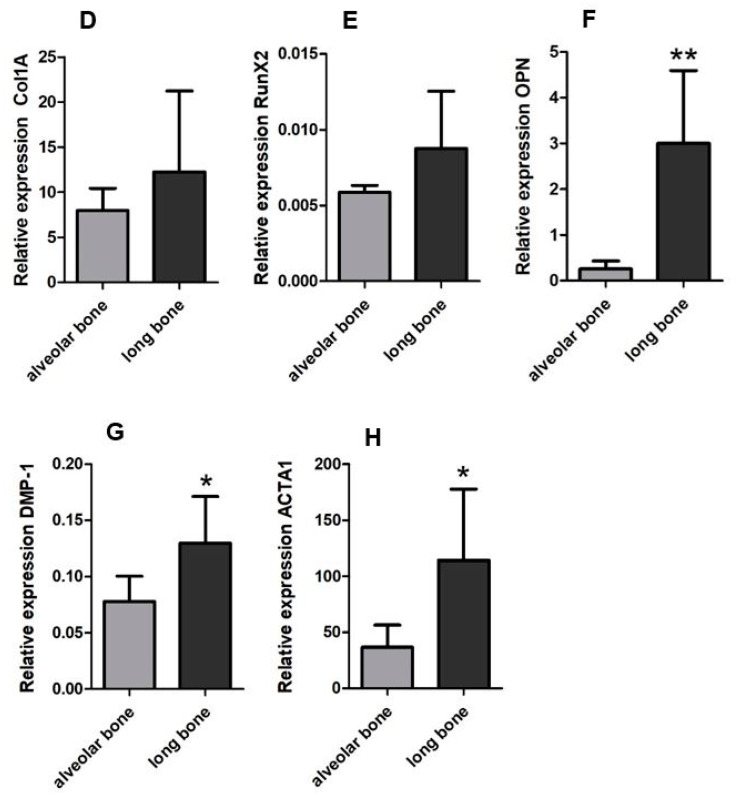
Basic differences between the cells from the alveolar bone and long bones. Light microscopy of (**A**) the alveolar bone cells and (**B**) the long bone cells at day 7. (**C**) DNA content, as a measure of the number of cells at a given time point, of both cells derived from alveolar bone and the long bone at day 0, day 7, day 14 and day 21. To gain insight into the osteogenic lineage of both cell types, the gene expression of osteogenic genes was assessed at day 0 (**D**–**H**) The relative expression of both (**D**) *Col1a* and (**E**) *RUNX2* was comparable in both the alveolar bone cells and the long bone cells. The relative expression of (**F**) *OPN*, (**G**) *DMP-1* and (**H**) *ACTA1* was significantly higher at day 0, in long the bone cells compared to the alveolar bone cells. Significance for DNA was measured using 2-way ANOVA with Bonferroni post-tests. For the relative gene expression, significance was measured using a t-test. * *p* < 0.05, ** *p* < 0.01.

To identify the differences between the alveolar-derived bone cells and the long bone-derived cells, it is first of all important to know the baseline characteristics of the cells derived from both skeletal sites. We tested the differences in appearance, proliferation, and bone markers. In Figure 1A,B, representative micrographs of the cells derived from bone harvested at both sites, cultured on tissue culture plastic on day 7, are shown. At confluence, alveolar bone cells (Figure 1A) had a fibroblastic appearance, while the appearance of long bone cells was more cuboidal (Figure 1B). The proliferation of the cells was measured with the total DNA content (Figure 1C). At both day 14 and 21, there was significantly more DNA in the alveolar bone samples, indicating the higher proliferation for the cells derived from the alveolar bone. At day 14, there was 2.9-fold more DNA in the samples derived from alveolar bone cells compared to the long bone cells, and at day 21 there was 1.6-fold more DNA in the alveolar bone samples compared to the long bone samples. The gene expression of several bone markers (Figure 1D–H) was measured at day 0. The relative expression of extracellular matrix protein *Collagen type I* (*Col1A*) (Figure 1D) and the differentiation marker *Runt-related transcription factor 2* (*RUNX2*) (Figure 1E) seemed higher in the cells derived from the long bones but this was not significant. In contrast, all the relatively late markers, *OPN* (Figure 1F), *Dentine matrix protein 1* (*DMP-1*) (Figure 1G) and *ACTA1* (Figure 1H) were significantly higher expressed in long bone cells than in alveolar bone cells. 

### 2.2. Cells Derived from Long Bone Have a Higher Expression of Late Osteogenic Markers Compared to Alveolar Bone Cells

**Figure 2:**
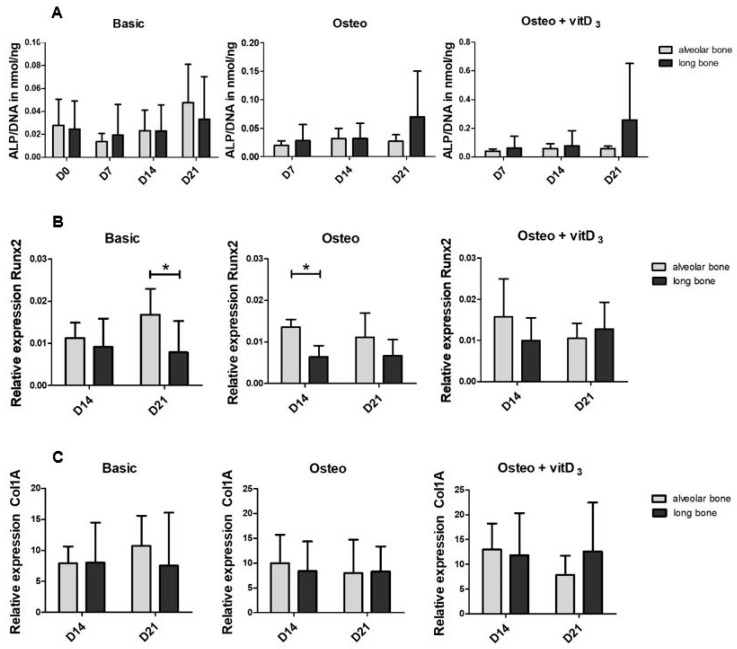

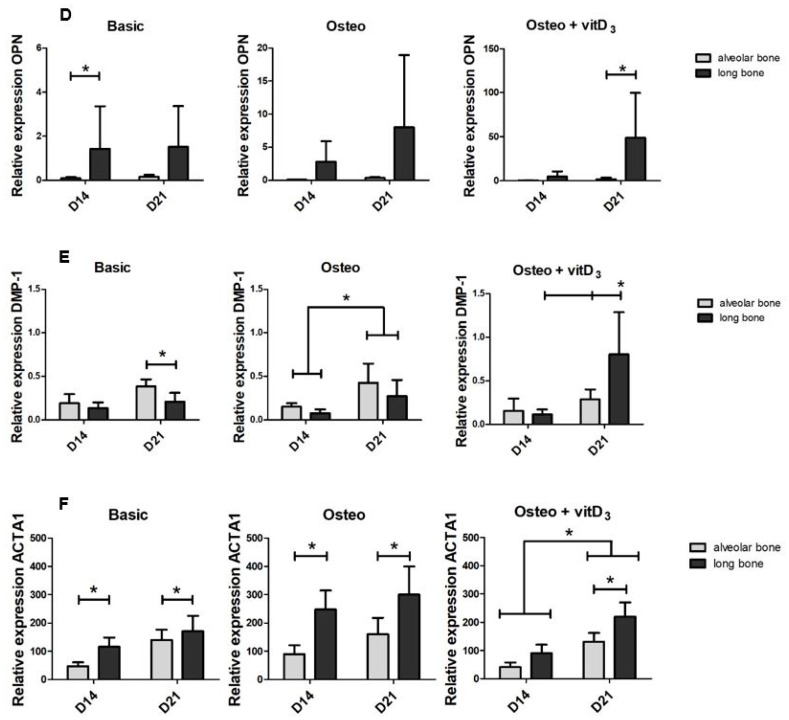
The osteogenic potential of the alveolar bone cells and the long bone cells in the osteogenic medium and the osteogenic medium supplemented with vitD_3_. Graphs depict the relative expression in the basic, osteogenic, and the osteogenic + vitD_3_ conditions of the cells derived from the alveolar bone and the long bone. Since the osteogenic differentiation is usually assessed at 14 and 21 days, the expression was measured at day 14 and day 21. (**A**) Cellular alkaline phosphatase (ALP) expression corrected for the DNA levels. (**B**) *RUNX2* expression. (**C**) *Col1a* expression. (**D**) *OPN* expression. (**E**) *DMP-1* expression. (**F**) *ACTA1* expression. Significance was measured using 2-way ANOVA with Bonferroni post-tests. * *p* < 0.05.

Having determined the basic differences between alveolar bone cells and long bone cells, we then assessed the osteogenic potential of both cell types. We tested this potential with an osteogenic medium and an osteogenic medium supplemented with vitD_3_. Cellular ALP activity levels (Figure 2A) were similar in alveolar bone and the long bones under all conditions. The differentiation marker *RUNX2* was under basic circumstances, significantly higher expressed by the alveolar bone cells than by long bone cells at day 21. This difference was not present when vitD_3_ was added. VitD_3_ upregulated the gene expression of *RUNX2* of long bone cells (Supplementary Materials Figure S1A). The relative expression of *Col1a* was similar in all conditions (Figure 2C). *OPN* expression appeared higher in the long bone samples compared to the alveolar bone samples for all conditions, but was only significantly higher at day 14 in a basic condition and day 21 in the condition with vitD_3_. Over time, the expression of *DMP-1* significantly increased in alveolar bone cell cultures in basic condition (Figure 2E). The osteogenic medium led to a significant increase in *DMP-1* expression when comparing day 14 with day 21, in both alveolar bone cells and long bone-derived cells. The expression of *DMP-1* was upregulated by the supplementation of vitD_3_ in long bone cells, specifically at day 21 (Supplementary Materials Figure S1B). At this time point, it was also significantly higher than the *DMP-1* expression in alveolar bone cells. The *ACTA1* expression was higher in the long bone cells than in the alveolar bone cells both in the basic and osteogenic conditions (Figure 2F). The addition of vitD_3_ seemed to lower the expression for both cell types at day 14. At day 21, the expression was significantly upregulated for both cell types and there was a significant difference between the alveolar bone cells and the long bone cells. The calcium deposits as a measure for mineralization were assessed using alizarin red staining. At day 21, there were no calcium deposits visible in both cell types (Supplementary Materials Figure S1C).

### 2.3. Communication Genes in Osteogenic Conditions of Alveolar Bone Cells and Long Bone Cells

**Figure 3:**
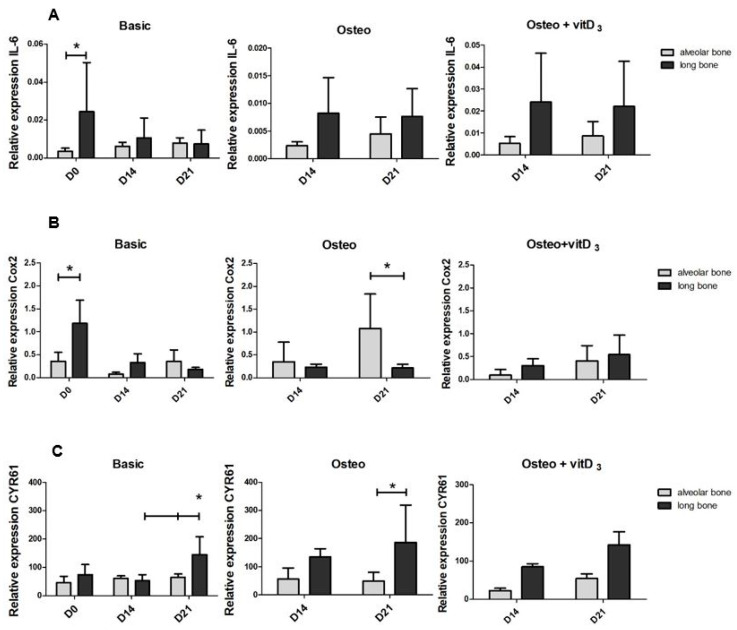
Differences in the expression of mRNAs associated with communication in the osteogenic conditions of the alveolar bone cells and the long bone cells. Graphs depict the relative expression in the basic, osteogenic, and the osteogenic + vitD_3_ conditions of the cells derived from the alveolar bone and the long bone. Relative expression was measured at day 0, day 14, and day 21. (**A**) Relative expression of *IL-6.* (**B**) Relative expression of *COX2*. (**C**) Relative expression of *CYR61*. The significance of the relative gene expression was measured using a 2-way ANOVA with Bonferroni post-tests. * *p* < 0.05.

After assessing the osteogenic potential for both cell types, we next wanted to determine if the alveolar bone cells and long bone cells differred in the expression of molecules that are associated with communication. The expression of *Interleukin 6* (*IL-6*) (Figure 3A), *Cyclo-oxygenase-2* (*COX2*) (Figure 3B), and *Cysteine-rich angiogenic inducer 61* (*CYR61*) (Figure 3C) were measured. *IL-6* can be secreted by osteoblasts to stimulate the formation of osteoclasts, but *IL-6* also stimulates the differentiation of osteoblasts. At day 0, the relative expression of *IL-6* was significantly higher in long bone cells (Figure 3A). The osteogenic medium influenced the expression of *IL-6* differently in the two cell types. In the alveolar bone cells, it seems to be inhibited while it induced the expression in long bone cells. The expression was significantly higher in the long bone cells compared to the alveolar-derived bone cells (Supplementary Materials Figure S2A). The supplementation of vitD_3_ induced the expression of *IL-6* both in the cells derived from alveolar bone and long bone, but there was no difference in the gene expression between the cells (Supplementary Materials Figure S2A). *COX2* is needed for normal fracture healing [17]. The relative expression of *COX2* was 3.3-fold higher in the long bone cells at day 0. At day 14 and day 21, the expression levels were similar between long bone and alveolar bone cells. The osteogenic medium upregulated the *COX2* expression, particularly in alveolar bone cells, leading to a 5-fold difference in the expression between the two cell types at day 21. Interestingly, supplementation with vitD_3_ induced the expression of *COX2* in long bone cells, especially on day 21 (Supplementary Materials Figure S2B). Besides being a matrix protein, *CYR61* promotes osteogenic differentiation [18]. Long bone cells expressed more *CYR61* than alveolar bone cells, in almost all conditions, but only significantly at day 21 under basic and osteogenic conditions.

### 2.4. Long Bone Cells Induce More Osteoclast Formation than Alveolar Bone Cells

**Figure 4:**
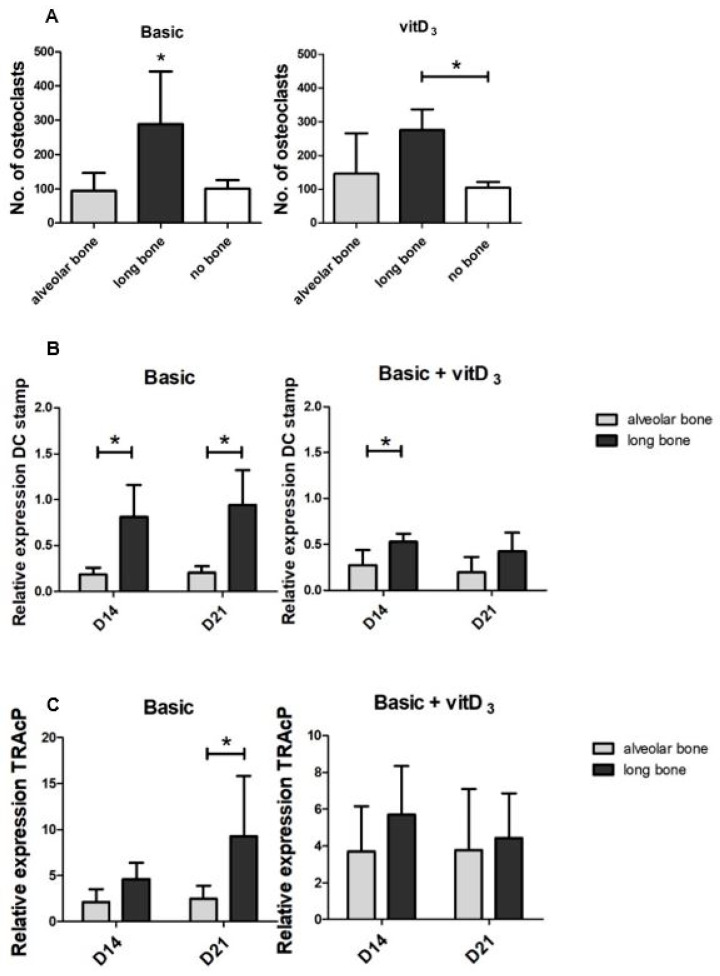
Osteoclastogenic potential of the alveolar bone cells and the long bone cells in the basic medium and the basic medium supplemented with vitD_3_. Osteoclastogenic potential of the co-cultures with the PBMCs and long bone cells and the PBMCS and alveolar bone cells. Graphs depict the basic medium and the basic medium + vitD_3_. (**A**) The total number of osteoclasts induced by athe lveolar bone cells, long bone cells, and the PBMCs alone at 21 days. Differentiation was further assessed with qPCR for osteoclast-specific genes (**B**,**C**). (**B**) The relative expression of DC-STAMP. (**C**) Relative expression of TRAcP. Significance for the number of osteoclasts was measured using a one-way ANOVA with a Tukey post-hoc test and the significance of the relative gene expression was measured using 2-way ANOVA with Bonferroni post-tests. * *p* < 0.05.

Besides the osteogenic potential of the both cell types, we also assessed the osteoclastogenic potential of the cell types. After 21 days of culture, the total number of osteoclasts was counted (Figure 4A). A distinction was made between the osteoclasts with 3–5 nuclei and 6+ nuclei, but only the total number of osteoclasts was shown since low numbers of larger osteoclasts were counted. On average, 19.2 ± 20.4 large osteoclasts were found in long bone, 2.9 ± 4.3 in alveolar bone, and 0.8 ± 0.5 in peripheral blood mononuclear cells (PBMCs) only (no bone) cultures. There were significantly more osteoclasts induced by long bone cells than by alveolar bone cells and PBMCs only, as a control condition. In the condition with vitD_3,_ the same trend is visible although not significantly. Interestingly, similar numbers of osteoclasts were counted in alveolar bone-PBMC co-cultures as in the PBMCs alone control cultures. In this control condition, without bone-derived cells, this was 93.8 ± 51 and 100 ± 25 osteoclasts, respectively, and in the condition supplemented with vitD_3_, it was 145.4 ± 114.8 and 104.3 ± 17.1 osteoclasts, respectively. In line with the increased osteoclast counts in long bone-PBMC co-cultures, the relative expression of osteoclast marker *DC-STAMP* was significantly higher in long bone at day 14 and day 21 (Figure 4B). At day 14, the average expression was 4.3-fold higher and 4.6-fold higher at day 21. In the condition with vitD_3,_ the expression of *DC-STAMP* was higher in long bones, which was significant at day 14. The relative expression of *Tartrate-resistant acid phosphatase* (*TRAcP*) was significantly higher in the control condition in the long bone cells at day 21 (Figure 4C). In the condition with vitD_3,_ there were no differences.

### 2.5. Expression of Pro-Osteoclastogenesis Proteins and mRNAs

**Figure 5:**
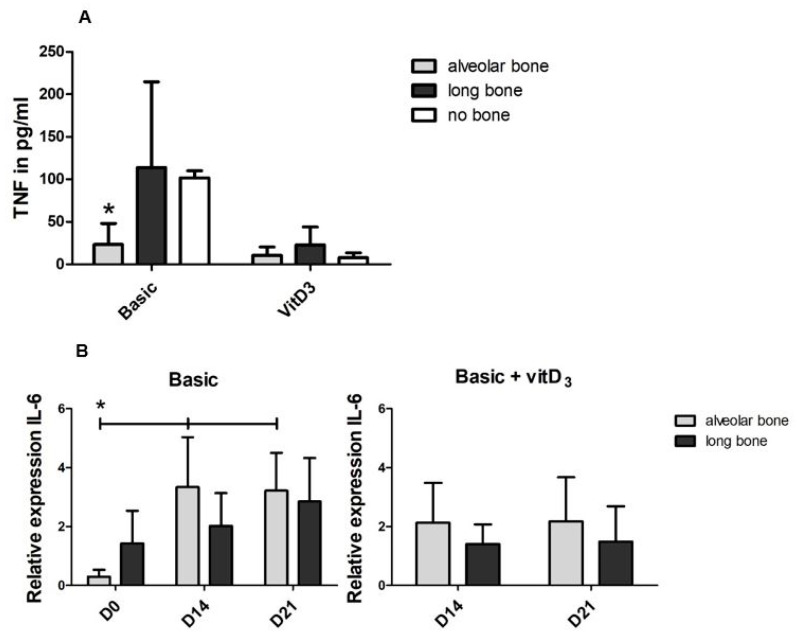

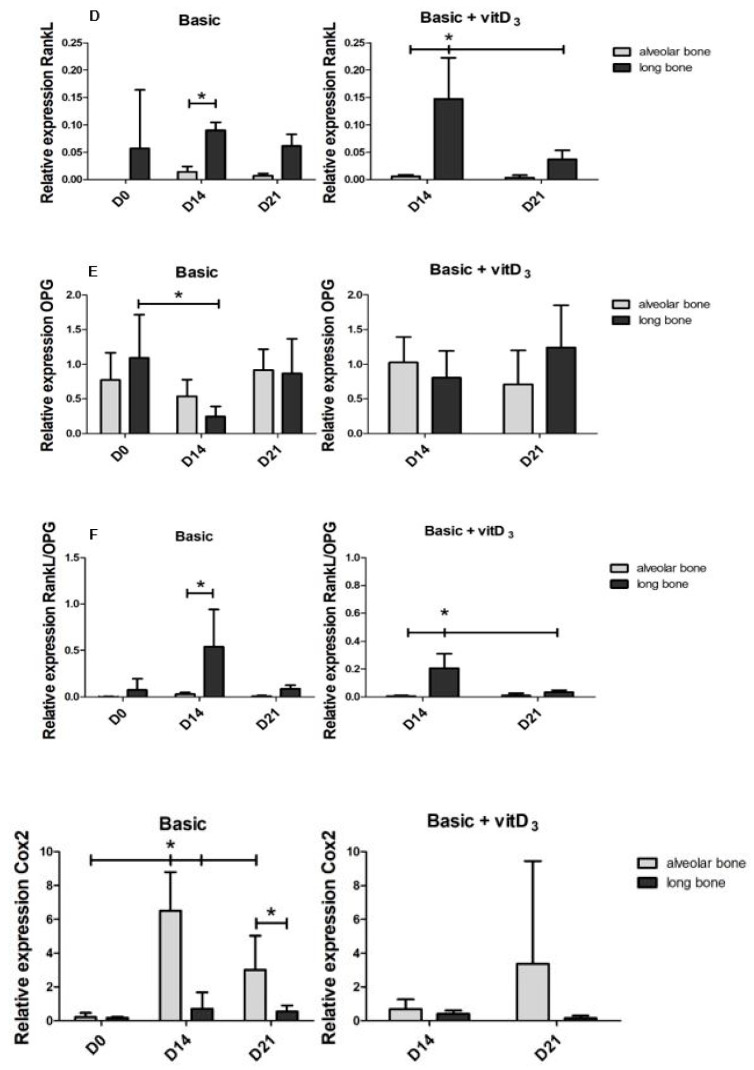
Differences in the proteins and the genes involved with osteoclastogenic differentiation in the alveolar bone cells and the long bone cultures. Graphs depict the basic medium and the basic medium + vitD_3_ (**A**) Concentration of TNF-α in media at day 7. (**B**) Relative expression of *IL-6*. (**C**) Relative expression of *RankL*. (**D**) Relative expression of osteoprotegerin *(OPG)*. (**E**) The ratio *RankL/OPG* gives an indication for osteoclast activation. (**F**) Relative expression of *COX2*. Significance was measured using a 2-way ANOVA with Bonferroni post-tests. * *p* < 0.05.

To explain the higher numbers of osteoclasts formed during the co-culture with long bone cells, compared to the alveolar bone-derived cells, we then assessed TNF-α and Interleukin-1 beta (IL-1β) in the supernatant of the osteoclastogenesis experiments (Figure 5A). The concentrations were determined at day 7, the time point with the peak expression of TNF-α [19]. The concentration of TNF-α was significantly lower in the co-cultures with alveolar bone cells (24 pg/mL) than in the co-cultures with long bone cells (114 pg/mL) and PBMSc alone (102 pg/mL). The concentration seemed lower when vitD_3_ was added in all groups. The concentration of IL-1β was too low to detect in all conditions. The relative expression of *IL-6* in the basic condition increased after day 0 for both cell types (Figure 5B). In the condition with vitD_3_, no differences were present. The expression of *IL-6* in the osteoclastogenesis experiments was on average 369-fold higher at day 14 and 395-fold higher at day 21 than the *IL-6* expression in the osteogenesis experiments under basic culture condition. The relative expression of *RankL* was higher in the long bone cells than in alveolar bone cells, significantly so at day 14 (Figure 5C). Although adding vitD_3_ caused a significantly higher expression of *RankL* in the long bones cultures at day 14 compared to the alveolar bone co-cultures, it decreased at day 21. Osteoprotegerin (*OPG*) is an important negative regulator of osteoclast differentiation. Under basic conditions, its expression decreased at day 14 in long bone-PBMC co-cultures (Figure 5D). Supplementation with vitD_3_ did not lead to a difference between the long bone cells and alveolar bone cells. The ratio of *RankL* and *OPG* is generally used as an indication of osteoclast differentiation. At day 14, this ratio is significantly higher (19.6-fold) in the long bone cells than in alveolar bone cells (Figure 5E), concomitant with the increased osteoclastogenesis by long bones. VitD_3_ lowers this ratio. In alveolar bone cells, the expression of *COX2* was on average 9.2-fold higher than in long bones at day 14 and 5.6-fold at day 21 (Figure 5F). At day 14, the supplementation of vitD_3_ led to the inhibition of *COX2* expression and induced its expression in long bone cells (Supplementary Materials Figure S3).

## 3. Discussion

In this study, we compared the ability of bone cells derived from human alveolar and long bones to differentiate towards mature bone forming osteoblasts, and their ability to stimulate osteoclast formation. These experiments were performed in the presence and absence of vitD_3,_ since vitD_3_ could stimulate differentiation when incorporated in tissue-engineering constructs as we have recently proposed [16]. We found that under basic conditions without VitD_3,_ long bone cells seemed to be more differentiated than alveolar bone cells, both at baseline and over time. Furthermore, long bone-derived cells induced more osteoclasts than alveolar bone cells. Taken together, our results suggest that long bone-derived cells are more differentiated, more actively communicate with osteoclast precursors, and have a stronger osteoclastogenic potential than alveolar bone cells.

Although the cells derived from both skeletal locations were isolated using the same protocol, the cells differed in their basic appearance, proliferation, and expression of bone markers. The long bone cells in our study had a more cuboidal appearance at day 7, while alveolar bone cells had a fibroblastic appearance. This indicates that long bone cells have a more osteoblastic appearance from the start, as osteoblasts are cuboidal shaped cells. In vivo, this cuboidal appearance is required for tightly connecting to neighboring cells, needed for in the consort deposition of osteoid [20,21]. Alveolar bone-derived cells proliferate faster than the cells derived from long bones, and this might have to do with the higher bone turnover in the jaw [22] and is also in line with a less differentiated state. Several late to very-late bone differentiation markers (*OPN, DMP-1,* and *ACTA1*) were higher expressed in long bone cells than in alveolar bone cells (Figure 1), underscoring their more mature, differentiated phenotype. *OPN* is a protein used as a marker for osteogenic differentiation. *DMP-1* is a bone mineralization marker and is expressed in early and mature osteocytes [23]. Furthermore, *ACTA1* is highly expressed in osteocytes [24]. Taken together, our data suggest that long bone cells have a more late- osteoblastic/preosteocyte-like phenotype than alveolar bone-derived cells.

Besides the basic differences, we also compared the osteogenic differentiation of both cell types. Again, the cells derived from long bones showed a higher degree of differentiation, as *OPN* and *ACTA1* expression was higher and vitD_3_ induced the expression of *RUNX2* and *DMP-1* more in long bone cells than in alveolar bone cells (Figure 2). This is in contrast with the stronger osteogenic potential described by Aghaloo et al. in mandibular MSCs compared to the MSCs from long bones [25]. This difference might lie in the cell source; they used cells derived from rats, we used cells from human donors. Rats continue to grow throughout their lifespan, whereas humans do not. Moreover, alveolar bone-derived cells might differ from the cells derived elsewhere from the mandibula. Differences in the effect of vitD_3_ on osteoblasts were also found in murine osteoblasts, where vitD_3_ had no effect on the osteoblast derived from long bones while inhibiting mineralization by calvarial osteoblasts [26]. In this study, both the cells derived from alveolar bone and long bone did not mineralize at day 21. Ruppeka-Rupeika et al. showed that alveolar bone-derived cells are capable of forming mineralizing nodules at day 21 [27]. One of the differences between the study by Ruppeka-Rupeika and our study, is the serum that was used to culture the cells. They only used FC1, while for the current comparison we used a combination of FC1 and FC3 for both cell types, since in our laboratory, the alveolar bone cells were routinely cultured in FC1 and the long bone cells in FC3. This difference indicates that the lack of nodule formation in both alveolar bone cells and long bone cells in the present study might be a result of the serum used. In any case, the cells from both origins showed an equal lack of bone nodule formation. 

We also investigated the osteoclastogenic potential of the cells derived from alveolar bone and long bone. Long bone-derived bone cells stimulated the formation of osteoclasts, while alveolar bone-derived cells did not, since numbers were comparable to the control condition without the bone cells added (Figure 4). Jaw and long bone-derived cells from the bone marrow of mice have different osteoclastogenic potential, mostly due to the differences in the cellular composition of the bone marrow [11]. This might also be true for the population of cells isolated by the outgrowth of cells. In line with the increased osteoclast formation induced by long bone cells is the higher expression of *RANKL*, TNF-α, and *IL-6* in long bone-PBMC co-cocultures, as well as in the increased *RANKL/OPG* ratio that favors osteoclast formation. Moreover, DC-STAMP is needed for cell–cell fusion [28] and was significantly higher expressed in the long bone-PBMC co-cultures in basic condition, supporting our finding that the cells derived from long bones induced the formation of more osteoclasts. Interestingly, the supplementation of vitD_3_ seemed to downregulate the expression of DC-STAMP in both co-cultures of PBMCs with long bone cells and with alveolar bone cells (Supplementary Materials Figure S4), which correlates with a lower number of osteoclasts counted in the condition with vitD_3_.

*COX2* expression was significantly higher expressed in alveolar bone cells. *COX2* is very important for fracture healing and plays a role in both osteoblast and osteoclast differentiation [29]. The inhibition of COX2 inhibits osteoclast formation [30], and this would suggest that the higher expression of *COX2* would induce osteoclast formation, which is in contrast with our data.

VitD_3_ is a biological component used in bone regeneration because it promotes the osteogenic differentiation of hMSCs by the upregulation of ALP and enhancing mineralization [31]. In the present study, the addition of vitD_3_ did not upregulate the cellular ALP, neither in alveolar bone cells nor in long bone cells. This might be because these cells are already in the osteogenic lineage and ALP is in general an early marker. Supplementation with vitD_3_ did enhance the expression of other osteogenic markers, such as *OPN* and *DMP-1*. VitD_3_ affects osteoclast formation by upregulating *RANKL* expression in human SaOS2 osteoblastic cells [32]. In contrast, supplementation with vitD_3_ to our human primary osteoblasts did not upregulate *RANKL* expression, and thereby did not enhance osteoclast formation. 

Our results are measured in the absence of mechanical loading, which it not representative for the in vivo situation, where in both the jaw as in the tibia, mechanical loading is present. Mechanical loading is known to have an enhancing effect on osteoblasts and a restrained effect on osteoclasts [33]. Adding mechanical loading to the cells would be interesting for further research on the differences in skeletal sites.

Due to donor to donor variability, some results had high SDs, which is observed more often when using human primary cells from multiple donors [16]. This high variance is inevitable, due to a higher degree of genetic differences between human individuals when compared to e.g., cells from inbred mouse or rat strains. Despite the variance between donors, many striking differences were observed in the present study. On the one hand, we would like to advocate such a comparison using cells from actual human donors, since it is likely closer to clinical reality when compared to using laboratory animals. On the other hand, and unfortunately, the cells derived from alveolar bone were not from the same donor as the cells derived from long bones, which would have made this study stronger. However, we can still see clear statistically significant differences between the cells derived from alveolar bone and long bone, which suggests that the two populations of cells are different. Many results were confirmed using different techniques, for instance, the osteoclasts were higher in long bone cultures, which was confirmed with the qPCRs of osteoclast genes expressed in these cultures.

In conclusion, the results of this present study showed that the cells derived from alveolar bone and long bone differ in their expression of osteogenic markers and their ability to induce osteoclast formation. We have to reject our hypothesis, as the results show that long bone cells instead of alveolar bone cells are more differentiated within the osteogenic lineage, and have increased osteoclastogenic potential. We show that cells from different skeletal sites, in this case, alveolar bone and long bone, are different and react differently to osteotropic agent vitD_3_. These differences show that skeletal sites give rise to site-specific differences between the cells and indicate, that for the regeneration of specific sites, such as the alveolar bone, more research on site-specific tissue-engineering constructs is necessary. 

## 4. Materials and Methods 

### 4.1. Bone Cell Cultures

In this study, we used two types of cells: cells derived from alveolar bone and long bones, in this case, the tibia. Both cell types were obtained by the outgrowth of cells from pieces of bone. In short, bone fragments were transported in Dulbecco’s modified Eagle’s medium (DMEM, Gibco, Paisley, UK) supplemented with 2% antibiotic antimycotic solution 100× (Sigma, St. Louis, MO, USA) and then cut into small pieces, washed with PBS and were incubated for 2 h in 2 mg/mL collagenase II (Sigma, St. Louis, MO, USA) in DMEM at 37 °C in a shaking water bath. The bone fragments were washed with a medium containing 10% fetal calf serum (FCS) and transferred to 25 cm^2^ flasks. Bone fragments were cultured in DMEM with 1% antibiotic antimycotic solution 100× and 10% FCS. When the cells reached confluency, the cells were harvested using 0.25% trypsin and 0.1% EDTA in PBS. For each cell type, we used cells from 5 different donors. Cells were not from the same donors and are from passage 2–4. The donors for the long bone cells were all female and were between 69-86 years of age. Of the alveolar bone cells donors, two were male and three were female with an age between 37 and 60. 

### 4.2. Alveolar Bone Cells

Cells were derived from human interdental alveolar crest bone. Patients who were referred for multiple adjacent tooth extractions and immediate denture placement were asked to participate. Interdental alveolar crestal bone was regarded as surgical waste since this bony rim has to be smoothened before wound closure. Treatments took place at the Department of Oral and Maxillofacial Surgery at the hospital OLVG in Amsterdam, The Netherlands. Prior to the treatment, written informed consent was signed by the patient, and the study protocol was approved by the research ethics committee of the OLVG (protocol-ID: WO17.194).

### 4.3. Long Bone Cells

Cells were derived from surgical waste from *human* bone from the knee, after surgery performed at the VU University Medical Center, Amsterdam, The Netherlands. This in agreement with The Ethical Review Board of the VU Medical Center, Amsterdam, The Netherlands, under protocol number 2016/105.

### 4.4. Osteogenesis Experiments

For the osteogenesis experiments, cells either derived from long bone or alveolar bone were seeded at 3 × 10^4^ cells/well in a 48-well plate and cultured in a basic medium, one day before the start of the experiment (day−1). At day 0, the medium was refreshed and the cells were cultured in basic medium, osteogenic medium, or osteogenic medium supplemented with vitD_3_. The basic culture medium consisted of the D modification of Dulbecco’s modified Eagle’s medium (DMEM, Gibco BRL, Paisley, Scotland), 5% fetal clone I serum (FCI, HyClone, Logan, UT), 5% fetal clone III serum (FCIII, HyClone) and 1% Antibiotic Antimycotic Solution, 100× (Sigma, St. Louis, MO, USA). Osteogenic medium is a basic medium supplemented with 50 µM ascorbic acid-2-phosphate (Sigma, St. Louis, MO, USA) and 50 µM β-Glycerophosphate (Sigma). In some cultures, osteogenic medium was supplemented with 10 nM vitD_3_ (osteogenic + vitD_3_). Medium was refreshed twice a week for 3 weeks.

#### 4.4.1. Alkaline Phosphatase (ALP) Activity

Cells were lysed with 200 µL of Milli-Q water at day 0, day 7, day 14, and day 21 and frozen at ‒20 °C. Samples were collected by scraping after 3 freeze–thaw cycles for ALP measurement. ALP was measured using 4-nitrophenyl phosphate disodium salt at pH 10.3 as a substrate for ALP, a method described by Lowry [34]. Absorbance was measured at 405 nm with Synergy HT^®^ spectrophotometer. ALP levels were corrected for DNA content and expressed as µmol/ng. DNA was measured using a Cyquant cell proliferation assay kit (Molecular probes, Eugene, OR, USA) according to the manufacturers’ instructions.

#### 4.4.2. Mineralization

Cells were cultured for 21 days to test for the mineralization of the cells. All the samples were washed with deionized water and fixed with 4% formaldehyde for 10 min. After rinsing with deionized water, 300 µL of 2% Alizarin red S (Sigma-Aldrich) staining with a pH of 4.3 was added to each well. After a 15-min incubation, the cells were washed and air-dried. 

#### 4.4.3. Quantitative Polymerase Chain Reaction (qPCR)

At day 0, and after 14 days and 21 days of culture, the total RNA was isolated with TRIzol^®^ reagent (Invitrogen) following the manufacturer’s protocol. The quality and concentration of the RNA were measured using a Synergy HT^®^ spectrophotometer. Then, 750 ng RNA was reverse-transcribed to cDNA using the RevertAid™ First Strand cDNA Synthesis Kit 1612 (Fermentas, St. Leon-Rot, Germany) according to the manufacturer’s protocol. For the qPCR reaction, cDNA was diluted 5× and 1 µL was used, together with 3 µL PCR-H_2_O, 0.5 µL (20 µM) forward primer, 0.5 µL (20 µM) reverse primer and 5 µl LightCycler^®^ 480 SYBR Green I Mastermix (Roche Diagnostics, Mannheim, Germany). All the measurements with a Ct value higher than 36 were considered unreliable and discarded. The values of all the genes were normalized to hypoxanthine phosphoribosyl transferase (HPRT) or glyceraldehyde 3-phosphate dehydrogenase (GAPDH) following the comparative cycle threshold (Ct) method and presented as the mean relative fold expression (2^−ΔCt^). All the primer sequences for osteogenesis experiments are listed in Table 1. 

### 4.5. Osteoclastogenesis Experiments

To analyze the capacity of alveolar bone and long bone-derived cells to contribute to osteoclast formation, these cells were seeded at 1.5 × 10^4^ cells/well in a 48-well plate and allowed to spread overnight (day −1). The next day (day 0), 0.5 × 10^6^ peripheral blood mononuclear cells (PBMCs) were seeded on top. The PBMCs were isolated from the buffy coat (Sanquin, Amsterdam, The Netherlands) by a standard density gradient centrifugation with Ficoll-Paque [35]. Co-cultures of bone cells and PBMCs were cultured for up to 21 days in basic medium (same composition as the basic medium in the osteogenesis experiments) or the basic medium supplemented with 10 nM vitD_3_. The medium was refreshed twice a week. As a control, the PBMCs on plastic, also without the supplementation of RankL and the macrophage colony-stimulating factor (M-CSF), were used.

#### 4.5.1. ELISA

After 7 days of culture, the supernatant of the osteoclastogenesis plate was collected and the Enzyme Linked Immunosorbent Assays were performed with the DuoSet ELISA kits (R&D systems, Abington, United Kingdom) for the human TNF-α, and IL-1β, following the manufacturer’s protocol.

#### 4.5.2. Osteoclast Quantification

After 21 days of culture, the cells were fixed with 4% formaldehyde for 10 min and stained with leukocyte acid phosphatase kit (Sigma) to identify the TRAcP activity. The nuclei were stained with diamidino-2phenylindole dihydrochloride (DAPI). The whole culture well was analyzed for the number of osteoclasts. Osteoclasts were considered as TRAcP+ multinucleated cells (MNCs) containing three or more nuclei. Two groups were counted, cells with 3–5 nuclei and cells with more than 5 nuclei.

#### 4.5.3. qPCR

At day 0, and after 14 days and 21 days of culture, the total RNA was isolated with TRIzol^®^ reagent (Invitrogen) following the manufacturer’s protocol. The quality and concentration of the RNA were measured using a Synergy HT^®^ spectrophotometer. Then, 750 ng RNA was reverse-transcribed to cDNA using the RevertAid™ First Strand cDNA Synthesis Kit 1612(Fermentas, St. Leon-Rot, Germany) according to the manufacturer’s protocol. For the qPCR reaction, cDNA was diluted 5× and 1 µL was used, together with 3 µL PCR-H_2_O, 0.5 µl (20 µM) forward primer, 0.5 µL (20 µM) reverse primer and 5 µL LightCycler^®^ 480 SYBR Green I Mastermix (Roche Diagnostics, Mannheim, Germany). All the measurements with a Ct value higher than 36 were considered unreliable and discarded. The values of all the genes were normalized to HPRT or GAPDH following the comparative cycle threshold (Ct) method and presented as the mean relative fold expression (2^−ΔCt^). All the primer sequences for osteoclastogenesis experiments are listed in Table 2. 

### 4.6. Statistics 

Data were obtained from the cultures of 5 independent donors (*n* = 5) for each cell type. Data are presented as mean + standard deviation (SD). A t-test was conducted to test for the statistical differences between the alveolar bone cells and the long bone cells at day 0 (Figure 1). The differences between the groups in the number of osteoclasts are measured with a one-way ANOVA with a Tukey post hoc test. Statistical comparisons of the experiments with multiple time points were done using a 2-way ANOVA with Bonferroni post-tests. With time and ‘cell type’ used as the dependent variables, the *P* values of < 0.05 were considered significantly different. The analyses were performed using GraphPad Prism 5.0 (GraphPad Software, San Diego, CA, USA).

## Figures and Tables

**Table 1:** Primer sequences osteogenesis.

Gene (Human)	Forward Sequence	Reverse Sequence
*COL1*	5′ GCATGGGCAGAGGTATAATG 3′	5′ GGTCCTTTGGGTCCTACAA 3′
*RUNX2*	5′ CCAGAAGGCACAGACAGAAGCT 3′	5′ AGGAATGCGCCCTAAATCACT 3′
*OPN*	5′ TTCCAAGTAAGTCCAACGAAAG 3′	5′ GTGACCAGTTCATCAGATTCAT 3′
*DMP-1*	5′ TAGGCTAGCTGGTGGCTTCT 3′	5′ AACTCGGAGCCGTCTCCAT 3′
*ACTA1*	5′ CTGGCACCACACCTTCTACA 3′	5′ CACGCTCAGTGAGGATCTT 3′
*IL-6*	5′ ACAGCCACTCACCTCTTCA 3′	5′ ACCAGGCAAGTCTCCTCAT 3′
*COX2*	5′ GCATTCTTTGCCCAGCACTT 3′	5′ AGACCAGGCACCAGACCAAAGA 3′
*CYR6*	5′ TCCGAGGTGGAGTTGACGAGAA3′	5′ TTCACAAGGCGGCACTCAGG 3′
*HPRT*	5′ GCTGACCTGCTGGATTACAT 3′	5′ CTTGCGACCTTGACCATCT 3′
*GAPDH*	5′ TGGGTGTGAACCATGAGAAGTATG 3′	5′ GGTGCAGGAGGCATTGCT 3′

**Table 2:** Primer sequences osteoclastogenesis.

Gene (Human)	Forward Sequence	Reverse Sequence
*DC-STAMP*	5′ ATTTTCTCAGTGAGCAAGCAGTTTC 3	5′ AGAATCATGGATAATATCTTGAGTTCCTT 3
*Tracp*	5′ CACAATCTGCAGTACCTGCAAGGAT 3′	5′ CCCATAGTGGAAGCGCAGATA 3′
*IL-6*	5′ ACAGCCACTCACCTCTTCA 3′	5′ ACCAGGCAAGTCTCCTCAT 3′
*RankL*	5′ CATCCCATCTGGTTCCCATAA 3′	5′ GCCCAACCCCGATCATG 3′
*OPG*	5′ TGGAATAGATGTTACCCTGTGTG 3′	5′ GCTGCTCGAAGGTGAGGTTA 3′
*COX2*	5′ GCATTCTTTGCCCAGCACTT 3′	5′ AGACCAGGCACCAGACCAAAGA 3′
*HPRT*	5′ GCTGACCTGCTGGATTACAT 3′	5′ CTTGCGACCTTGACCATCT 3′
*GAPDH*	5′ TGGGTGTGAACCATGAGAAGTATG 3′	5′ GGTGCAGGAGGCATTGCT 3′

## Supplementary Materials

The following are available online at https://www.mdpi.com/1422-0067/21/14/5072/s1.

## Abbreviations

vitD_3_ 1.25(OH)_2_	vitamin D_3_
PBMCs	peripheral blood mononuclear cells
OPN	osteopontin
ACTA1	Actin alpha 1
TNF-α	Tumor necrose factor-alfa
DC-STAMP	dendritic cell specific transmembrane protein
RankL	Receptor activator of nuclear factor kappa-Β ligand
hMSCs	human bone marrow stromal cells
ALP	alkaline phosphatase
BMP-2	bone morphogenetic protein 2
Col1a	collagen type 1
RUNX2	Runt-related transcription factor 2
DMP1	Dentine matrix protein 1
IL-6	Interleukin 6
COX2	Cyclo-oxygenase-2
CYR61	Cysteine-rich angiogenic inducer 61
TRAcP	Tartrate-resistant acid phosphatase
IL-1β	Interleukin-1 beta
OPG	Osteoprotegerin
M-CSF	macrophage colony-stimulating factor
FCS	fetal calf serum
