# Supplementary material for: Cells Derived from Human Long Bone Appear More Differentiated and More Actively Stimulate Osteoclastogenesis Compared to Alveolar Bone-Derived Cells
Source: Int J Mol Sci. 2020 Jul 17;21(14):5072. doi: 10.3390/ijms21145072 (PMC7404058; doi:10.3390/ijms21145072)
Supplement: Supplementary file 1 [file ijms-21-05072-s001.pdf]

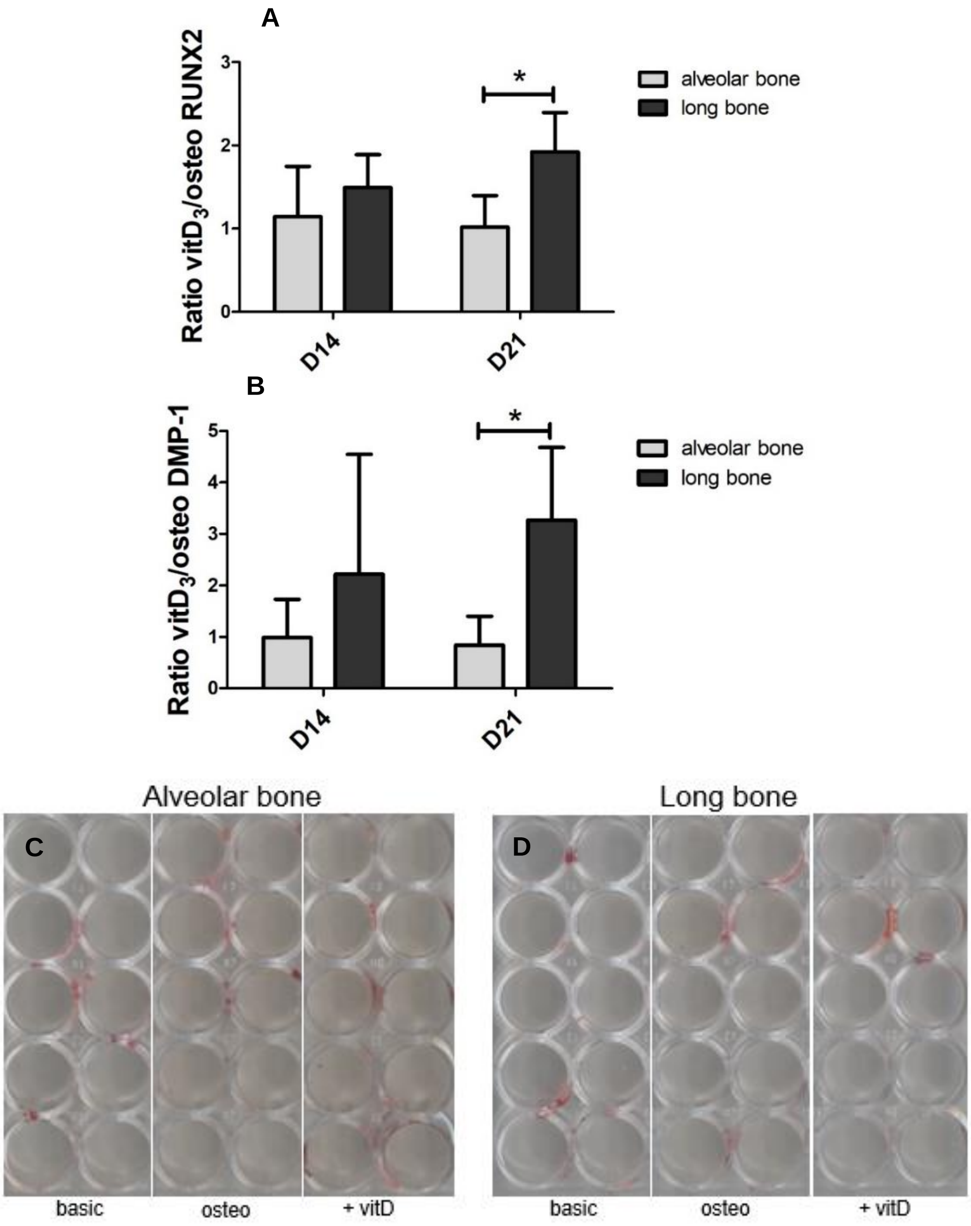

Figure S1. Graphs depict the osteogenic medium + vitD$_3$ over the osteogenic medium at day 14 and day 21. (**A**) Effect of vitD$_3$ on the *RUNX2* expression. vitD$_3$ had more effect on the long bone cells, which is significantly different on day 21. (**B**) Effect of vitD$_3$ on the *DMP-1* expression. vitD$_3$ had more effect on long bone cells, which is significantly different on day 21. (**C**) Alizarin red staining for the calcium deposits. No calcium deposits were visible for both the alveolar bone cells and the long bone cells. Significance was measured using a 2-way ANOVA with Bonferroni post-tests. * = $p < 0.05$.

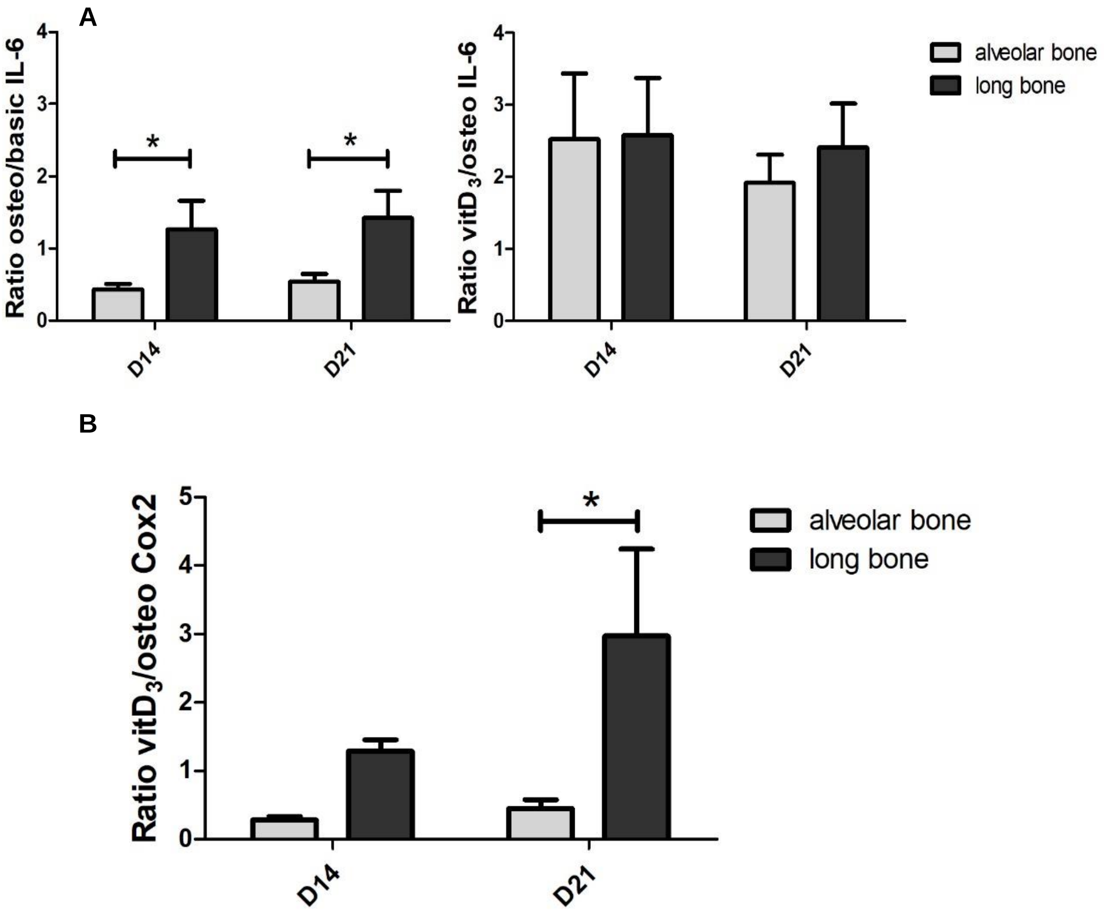

Figure S2. Graphs depict osteogenic medium over the basic or the osteogenic medium + vitD3 over the osteogenic medium. (**A**) Osteogenic medium significantly increased the expression of *IL-6* in the long bone cells. Supplementation of vitD3 upregulated the *IL-6* expression in both the alveolar bone cells and the long bone cells. (**B**) Effect of vitD3 on the *COX2* expression. VitD3 had more effect on the long bone cells, which is significantly different on day 21. Significance was measured using 2-way ANOVA with Bonferroni post-tests. * = $p < 0.05$.

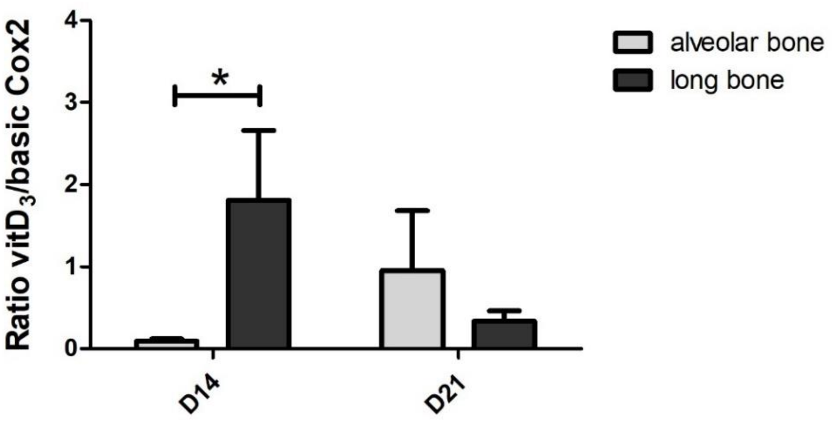

Figure S3. Graph depicts the basic medium + vitD3 over the basic medium. Effect of vitD3 on the *COX2* expression. On day 14, vitD3 significantly upregulated the *COX2* expression. Significance was measured using 2-way ANOVA with Bonferroni post-tests. * = $p < 0.05$.

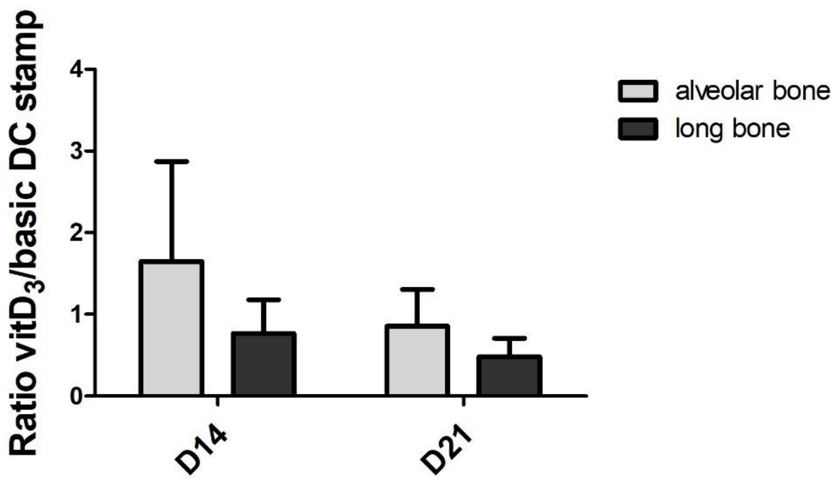

Figure S4. Graphs depict the basic medium + vitD_3 over the basic medium. (A) Effect of vitD_3 on the *DC stamp* expression. Significance was measured using 2-way ANOVA with Bonferroni post-tests.
